# CTD data over a repeated section in the Vema Channel

**DOI:** 10.1016/j.dib.2021.107211

**Published:** 2021-06-09

**Authors:** Eugene G. Morozov, Dmitry I. Frey

**Affiliations:** Shirshov Institute of Oceanology, Russian Academy of Sciences, Moscow, Russia

**Keywords:** CTD, Vema Channel, Abyssal water, Antarctic bottom water, Abyssal currents, South Atlantic

## Abstract

We present a CTD dataset of repeated sections across the Vema Channel in the South Atlantic approximately along 31°12′ S between longitudes 39°18.0 W and 39°30.0′ W. The Vema Channel is a narrow conduit for Antarctic Bottom Water across the Rio Grande Rise. The measurements at CTD stations (Conductivity-Temperature-Depth) across the Vema Channel were started by German scientists in 1991. In 2002, Russian scientists took part in this work and have been establishing stations across this standard section until recently. The data were collected using the Sea-Bird Electronics SBE-19 profiler. The data are presented in tabular format. The data are available at http://dx.doi.org/10.17632/hh4hhn6ny8.1.

## Specifications Table

**Table:** 

Subject	172 Oceanography
Specific subject area	Data of ten CTD-sections to the bottom across the Vema Channel at 31°12′ S from 2002 to 2020
Type of data	Tables
How data were acquired	The data were acquired by CTD profiling from the surface to the bottom using SBE-19 profiler from a ship that kept its position. Several CTD casts were made across the Vema Channel.
Data format	Raw data. The data are presented in the ASCII code as three columns pressure, temperature, salinity.
Parameters for data collection	Data of pressure, temperature, and salinity with a vertical step of 2 dbar.
Description of data collection	The data were collected by CTD-casts in the Vema Channel from the surface to the bottom using SBE-19 profiler. All data were converted to ASCII format
Data source location	31°12′ S - 31°14′ S, 39°16′ W - 39°40′ W in the South Atlantic
Data accessibility	The data are in a public repository: **Mendeley Data** http://dx.doi.org/10.17632/hh4hhn6ny8.1
Related research article	E.G. Morozov, D.I. Frey. V.A. Krechik, M.V. Kapustina, and M.N. Pisareva, Structure of the bottom water flow in the Vema Channel based on the measurements across the channel from the R/V “Akademik Sergey Vavilov”, Russian Journal of Earth Sciences, V. 21, ES3003, http://dx.doi.org/10.2205/2021ES000769, 2021

## Value of the Data

• We present repeated CTD data sections across the Vema Channel at 31°12′ S. This is one of very few long-term deep ocean time series.
• The data enables scholars of abyssal circulation to study the bottom flows.
• With Deep Argo coming online, any ship based CTD data is needed for reference and calibration of the float data.

## 1 Data Description

**Fig. 1:**
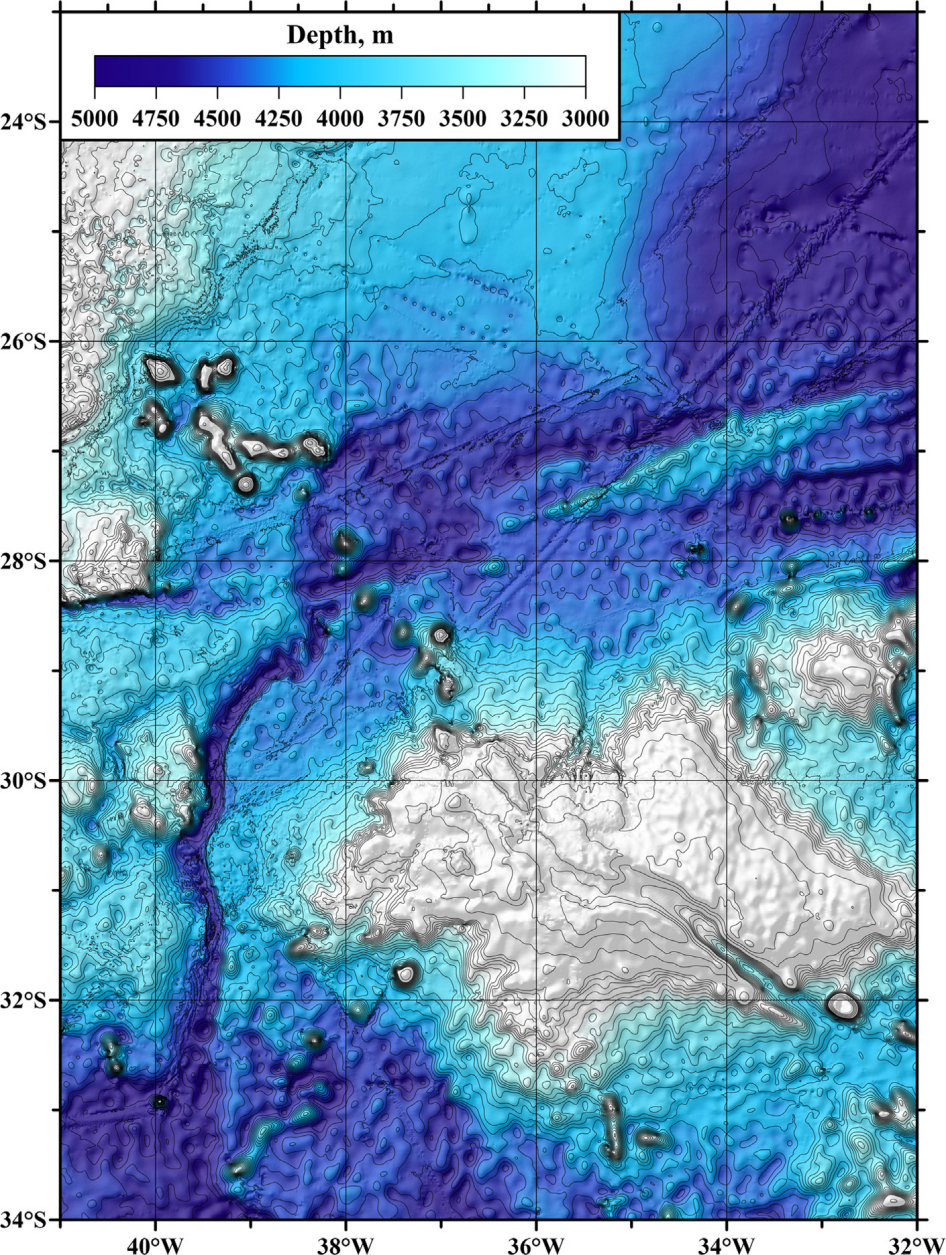
Chart of the bottom topography in the Vema Channel region. The bathymetry source is the GEBCO2019 database.

The Vema Channel connects the deep-water parts of the Argentine and Brazil basins. The densest and coldest Antarctic Bottom Water spreads through this channel. The depths of the Vema Channel exceed 4600 m, while the depths of the Santos Plateau (which the channel crosses) are about 4200 m. The channel is a narrow passage between two terraces. Its width at its narrowest part slightly exceeds 15 km. The mean transport of Antarctic Bottom Water across the section at 31°12′ S below potential temperature isotherm θ = 2.0 °C is estimated at 3.5 Sv. The mean velocities of the bottom flow are 30 cm/s, while the maximum reach 60 cm/s [3]. A chart of the region is shown in Fig. 1.

The channel was discovered in the 1950s and hydrological measurements began in 1972. The CTD-casts in the region of the standard section across the Vema Channel at latitude 31°12′ S has been made 25 times from 1991 to 2020. In some years, a section of 3–6 stations was carried out, sometimes only one station at 31°12′ S, 39°18′ W was occupied. Dates of sections of at least three stations occupied along 31°12′ S are presented in Table 1. In this paper we present only the data of ten Russian sections.

**Table 1:** CTD sections in the Vema Channel near the Vema Sill at 31°12′ S.

Time	Ship	Country
January-February 1991	RV *Meteor*	Germany
December 1992	RV *Meteor*	Germany
March 1996	RV *Meteor*	Germany
April 1998	RV *Meteor*	Germany
November 2002	RV *Akademik Ioffe*	Russia
November 2003	RV *Akademik Sergey Vavilov*	Russia
November 2004	RV *Akademik Ioffe*	Russia
March 2005	RV *Akademik Ioffe*	Russia
May 2005	RV *Polarstern*	Germany
October 2005	RV *Akademik Ioffe*	Russia
November 2006	RV *Akademik Ioffe*	Russia
April 2009	RV *Akademik Ioffe*	Russia
April 2017	RV *Akademik Sergey Vavilov*	Russia
October 2018	RV *Akademik Sergey Vavilov*	Russia
March 2020	RV *Akademik Mstislav Keldysh*	Russia

**Fig. 2:**
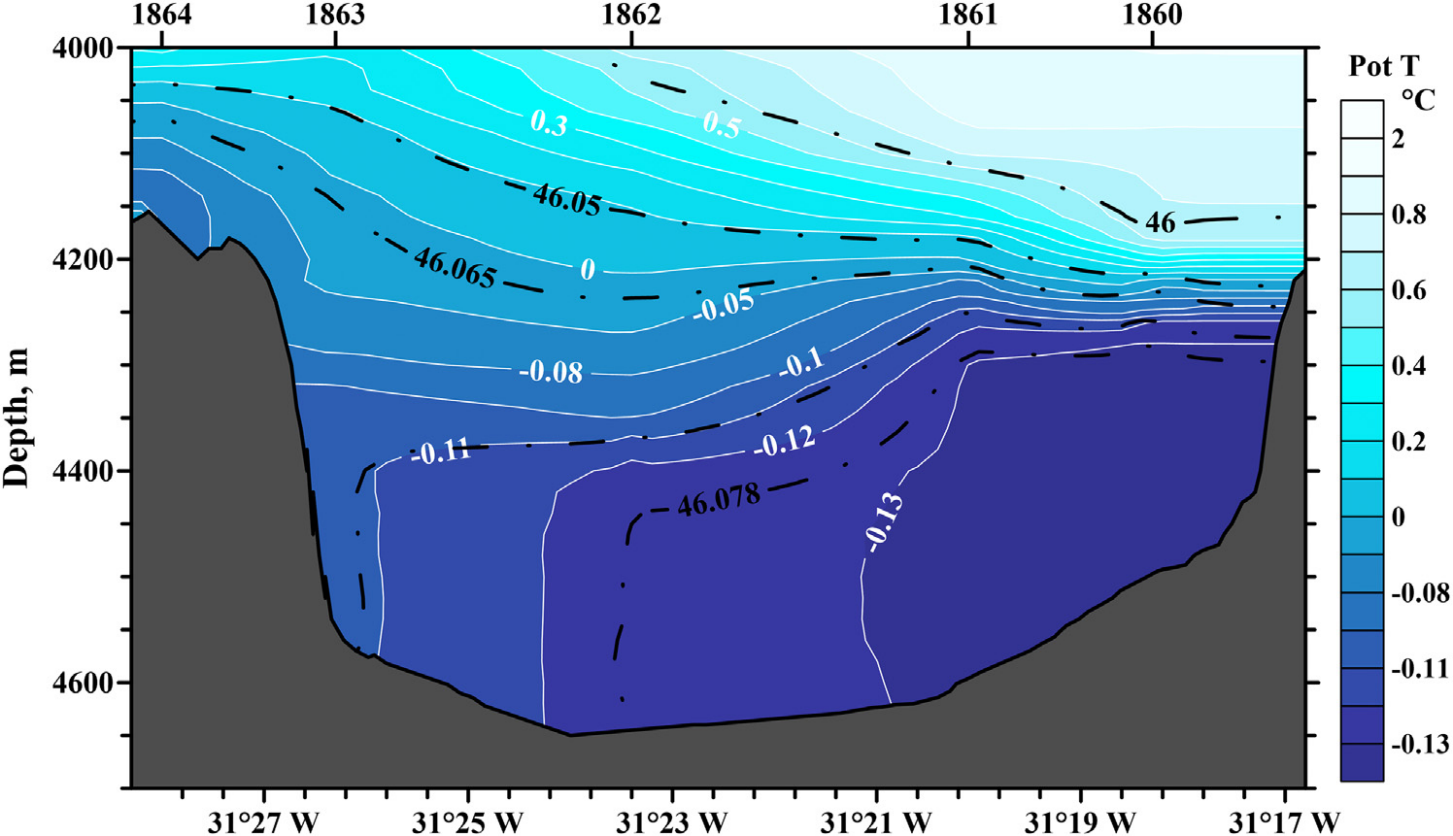
Section of potential temperature across the Vema Channel at 31°12′ S (deg C). Contour lines of density are shown as dashed-dotted lines. Sea bottom is shown in gray color.

The flow of Antarctic Bottom Water (AABW) in the channel is usually well mixed vertically in the bottom layer of 100–150 m. However, there is a horizontal stratification of the flow. Due to the Ekman frictional forces, the coldest core of the flow is usually displaced to the east, i.e., to the right wall of the channel in the direction of flow. This is in good agreement with the modeling data [1,2]. An example of potential temperature and salinity distribution over the section across the Vema Canal at 31°12′ S latitude is shown in Fig. 2.

Over a long period of time measurements on a standard section along 31°12′ S, a tendency to an increase in the potential temperature in the AABW core with time was found [4]. This conclusion was made based on repeated CTD casts at one point of measurements at 31°12′ S, 39°18′ W. Up to 1993, the potential temperature was -0.180°С, and the deviations did not exceed 0.01°С. Then, the potential temperature increased. After 2004, temperature fluctuations were observed between −0.120 °C and −0.130 °C, but according to observations until 2020, this trend continued.

The data were acquired by means of repeated CTD casts in the Vema Channel. The data supplemented to this publication includes 10 sections across the Vema Channel in the region 31°12′ S - 31°14′ S. A sill with gentle slopes crosses the Vema Channel at these latitudes. The deepest point on the crest of this sill is approximately 4614 m. The dates of sections and their longitudinal extensions are given in Table 2. Usually, the section was completed in one-three days. The data consist of the pressure, temperature, and salinity values with a vertical step of 2 dbar. The heading of each file includes station number, name of the ship, date and time of the cast, coordinates of the location, and depth of the ocean. The files are grouped in folders according to the time of each section.

**Table 2:** Stations of the sections along 31°12′ S -31°14′ S.

Dates	Numbers of stations	Latitude	Interval of western longitudes
Nov. 8–9, 2002	1059-1065	31°14′ S	39°17.1′- 39°28.0′
Nov. 11–13, 2003	1461, 1463-1468, 1470	31°14′ S	39°18.4′- 39°26.2′
Nov. 8–9, 2004	1630-1634	31°14′ S	39°18.2′- 39°26.3′
Mar. 30, 2005	1651-1655	31°12′ S	39°18.2′- 39°26.3′
Oct. 25–26, 2005	1703-1707	31°12′ S	39°18.3′- 39°26.4′
Nov. 11–12, 2006	1860-1864	31°12′ S	39°18.3′- 39°28.0′
Apr. 15–16, 2009	2074-2078	31°12′ S	39°18.4′- 39°24.9′
Apr. 5–6, 2017	2688-2695	31°12′ S	39°16.0′- 39°40.0′
Oct. 25–26, 2018	2718, 2720-2724	31°12′ S	39°18.0′- 39°29.0′
Mar. 28–29, 2020	6772-6780	31°12′ S	39°15.8′- 39°34.0′

## 2 Experiment, Materials and Methods

The sections across the Vema Channel were generally made approximately in one-three days. The sections were occupied along 31°12′ S or 31°14′ S. The small latitudinal variation of the section locations was made because in some years moorings were deployed in the region. Displacement of the sections was made to avoid collision with the moored instruments. The upcast and downcast were usually about 90 minutes long each. The ship was maintaining its position at a prescribed point. The instrument was lowered on a cable almost to the bottom in the profiling mode. The speed of lowering did not exceed 1 m/s. The cast was stopped based on the data of pinger and altimeter at a distance of 3–5 m above the bottom. The engineering codes were transformed to the raw data of pressure, temperature, and salinity using the calibration coefficients. The instrument was subjected to annual calibrations and revealed high stability. Data processing was performed using the standard Sea-Bird processing software: SeaTerm, SeaSave, SBE Data Processing (Data conversion, Filter, Align CTD, Loop edit, Derive, Bin average). The data are presented in the form of three columns: pressure, temperature, and salinity. Coordinates and time of the sections are in the header of files. In addition, the dates of the stations and the longitudinal interval are given in Table 2. Each section included from five to nine stations across the abyssal channel. The number of stations was determined by the available ship time. The presented data do not need any pre-processing and may be used directly.

## Ethics Statement

The data were collected by CTD profilers in the ocean.

The work does not include the use of human subjects.

The work does not include animal experiments.

The work does not include data collected from social media platforms.

The data belong to the authors of the article. They participated in the cruises and carried out the field work. Eugene Morozov was the principal scientist in all cruises.

## Author Contributions

Both authors participated in the field works, data collection and writing the paper.

## Declaration of Competing Interest

The authors declare that they have no known competing financial interests or personal relationships, which have, or could be perceived to have, influenced the work reported in this article.
